# Collagen VIα2 chain deficiency causes trabecular bone loss by potentially promoting osteoclast differentiation through enhanced TNFα signaling

**DOI:** 10.1038/s41598-020-70730-7

**Published:** 2020-08-13

**Authors:** Hai T. Pham, Vardit Kram, Qurratul-Ain Dar, Taishi Komori, Youngmi Ji, Payam Mohassel, Jachinta Rooney, Li Li, Tina M. Kilts, Carsten Bonnemann, Shireen Lamande, Marian F. Young

**Affiliations:** 1 Molecular Biology of Bones and Teeth Section, Department of Health and Human Services (DHHS), National Institute of Dental and Craniofacial Research (NIDCR), National Institutes of Health (NIH), Building 30 Room 5A509, Bethesda, MD 20892 USA; 2 Neuromuscular and Neurogenic Disorders of Childhood Section, Neurogenetics Branch, National Institute of Neurological Disorders and Stoke, Department of Health and Human Services, National Institutes of Health, Bethesda, MD 20892 USA; 3 Department of Pediatrics, University of Melbourne, Parkville, Australia

**Keywords:** Pathogenesis, Diseases, Metabolic bone disease, Mechanisms of disease, Osteoimmunology

## Abstract

Type VI collagen is well known for its role in muscular disorders, however its function in bone is still not well understood. To examine its role in bone we analyzed femoral and vertebral bone mass by micro-computed tomography analysis, which showed lower bone volume/total volume and trabecular number in *Col6α2*-KO mice compared with *WT*. Dynamic histomorphometry showed no differences in trabecular bone formation between *WT* and *Col6α2*-KO mice based on the mineral appositional rate, bone formation rate, and mineralizing perimeter. Femoral sections were assessed for the abundance of Tartrate Resistant Acid Phosphatase-positive osteoclasts, which revealed that mutant mice had more osteoclasts compared with *WT* mice, indicating that the primary effect of Col6a2 deficiency is on osteoclastogenesis. When bone marrow stromal cells (BMSCs) from WT and *Col6α2-*KO mice were treated with rmTNFα protein, the *Col6α2-*KO cells expressed higher levels of *TNFα* mRNA compared with *WT* cells. This was accompanied by higher levels of p-p65, a down-stream target of TNFα, suggesting that BMSCs from *Col6α2-*KO mice are highly sensitive to TNFα signaling. Taken together, our data imply that Col6a2 deficiency causes trabecular bone loss by enhancing osteoclast differentiation through enhanced TNFα signaling.

## Introduction

Bone is a highly dynamic tissue that depends on specialized cell types to regulate its formation and structural integrity. Bones are formed by the anabolic actions of osteoblasts. Osteoblasts share a close relationship with catabolic osteoclasts that resorb or digest bones. The coupled actions of bone resorption and subsequent bone formation is known as “bone turnover”, a process that replaces and renews bone tissue that has microdamage from impact or aging. An imbalance of bone turnover results in either osteopetrosis, which is excess bone matrix, or osteopenia, which is insufficient bone matrix. In both cases, the bone is fragile and prone to breakage. Identifying novel factors that affect the osteoblast-osteoclast relationship will improve our understanding of bone turnover and pave the way for future bone therapies.

Osteogenic cells are surrounded by mineralized connective tissue composed of collagen fibrils and non-collagenous proteins. These matrix components serve as nucleation sites for depositing the carbonate-rich apatite that provides strength and stiffness to the bone. Type I collagen is the most abundant collagen in bone and is composed of two α1(I) and one α2(I) chains that are assembled into a triple helical structure. These helices further aggregate to form fibrils outside the cell. The importance of type I collagen in bone is clearly illustrated by the clinical presentation of patients with mutations in type I collagen chains, or in the enzymes that regulate collagen maturation. These defects lead to the “brittle bone disease” also known as osteogenesis imperfecta (OI)^1,2^. While the functions of type I collagen in mineralized tissue have been well-investigated, the roles of other collagen types are unclear.

Type VI collagen is most often composed of three different α-chains (α1, α2, α3) with three other minor chains (α4, α5, and α6) found at low levels^3^. Mutations in any one of the three major chains of human type VI collagen cause Bethlem myopathy or Ullrich congenital muscular dystrophy (UCMD), diseases that present with mild to severe muscle weakness, respectively^4^. While type VI collagen is found in many musculoskeletal tissues, its functions in bone are just beginning to be investigated^5^. Assembly of the type VI collagen triple helix begins at the C-terminus^6^ and the triple helical monomer is flanked by two large multidomain globular regions^7^. Dimers are assembled from head-to-tail staggered monomers. Then, dimers align side-by-side to form tetramers, which are then secreted. Outside the cell, tetramers assemble end-to-end into microfibril structures that appear as “beads” when visualized by the electron microscope^4^. The importance of the ColVIα2 chain in the assembly, secretion and subsequent microfibril formation was elegantly highlighted by Tooley et al.^8^ who identified a UCMD patient with compound heterozygous mutations in α2 (VI). The mutant collagen was found to be retained intracellularly, preventing normal folding and microfibril assembly. In order to investigate the role of ColVI in bone and its mechanistic foundation, the skeletal phenotype of mice globally deficient in the ColVI α2 (Col6a2) protein was examined.

In this investigation, we show that collagen VI regulates trabecular bone mass in both the femur and spine by controlling the balance between bone formation and resporption. The reduced bone mass observed in *Col6α2-*KO mice arises from increased osteoclastogenesis with no apparent effect on bone formation. RNAseq analysis of mRNA extracted from the bones of *Col6α2-*KO mice showed deregulation of bone remodeling pathways, and in addition, pointed to a possible link to TNFα. Here we show that there is a direct interaction between Col6a2 and TNFα and in vitro, that Col6a2 attenuates TNFα-induced osteoclastogenesis to subsequently influence bone mass.

## Results

### ColVI protein and *Col6a2* mRNA expression is abundant in bone

**Figure 1:**
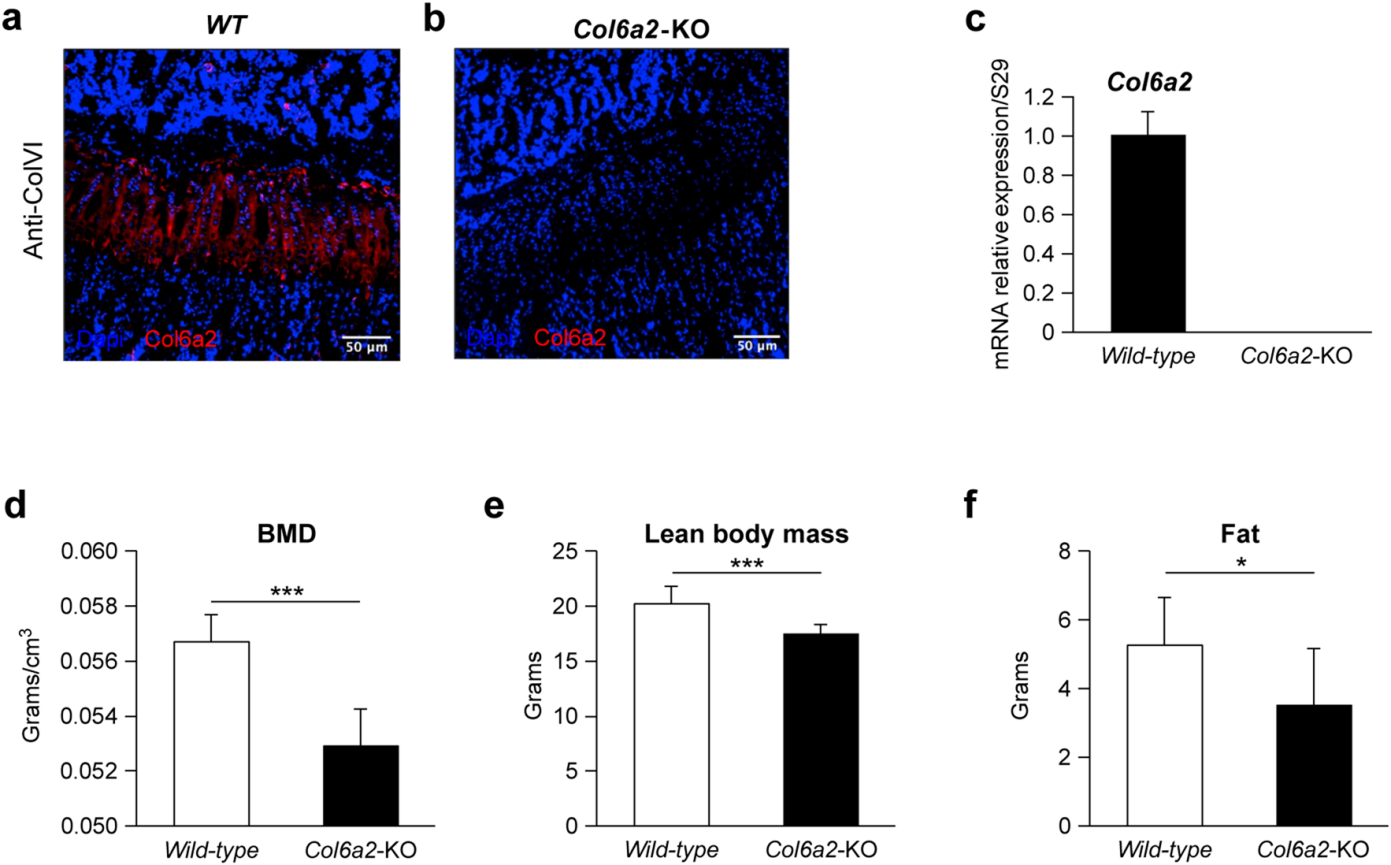
Immunoflourescence staining of ColVI in bone sections from one month-old mice *WT* and *Col6a2-*KO femur bones (**a**, **b**) representative experiment. Relative expression of *Col6a2* mRNA in *WT* and *Col6a2-*KO femur bones (**c**), N = 3 *WT* bones, N = 3 *Col6a2* bones with three technical replicates/bone. Whole body bone mineral density (BMD) (**d**), lean body mass (**e**) and fat content (**f**) in wild-type (white bar) vs Col6a2-KO mice (black bar), N = 6/genotype *p < 0.05, **p < 0.01, ***p < 0.001, ****p < 0.0001.

The expression of ColVI (all three chains) in bone was measured by immunofluorescence staining in bones from *WT* and *Col6a2-*KO mice. In 1 month-old *WT* mice, prominent expression was found in the hypertrophic cartilage layer that lies adjacent to newly formed bone in the primary ossification center (Fig. 1a). By contrast, no ColVI staining was seen in age and sex-matched *Col6a2-*KO mice (Fig. 1b). RT-PCR of *col6a2* mRNA extracted from whole *Col6a2-*KO bones showed complete depletion of Col6a2 mRNA compared with *WT* counterparts (Fig. 1b,c). The *Col6a2*-KO mice were slightly smaller than age (3-month) and sex (male) matched *WT* counterparts (not shown), and when subjected to DEXA analysis (Fig. 1e) showed lower whole-body Bone Mineral Density (BMD) than WT mice. The *Col6a2*-KO mice also had lower fat body mass and lower fat content compared to age (3-month) and sex (male) matched *WT* mice (Fig. 1f).

### Trabecular bone is reduced in *Col6a2-*KO mice

**Figure 2:**
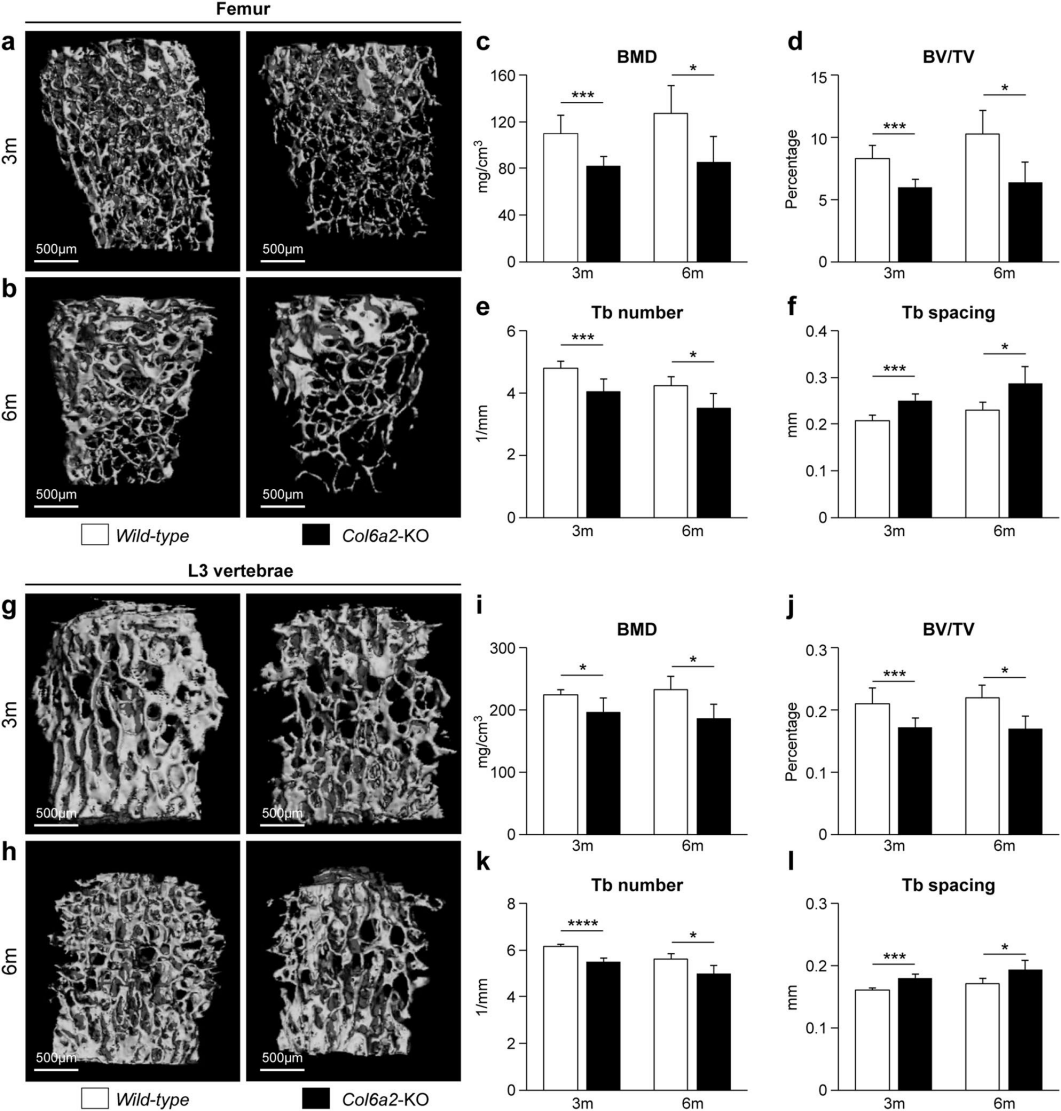
Low trabecular bone mass phenotype in *Col6a2*-KO mice vs *WT* mice. (**a**) 3D rendering of the distal femoral metaphyseal bone from 3 month-old (3 m) *WT* vs *Col6a2*-KO, representative images (**b**) and 6 month-old (6 m) mice. Representative images (**c**) Quantitative µCT analysis of Bone Mineral Density (BMD), (**d**) Bone Volume/Total Volume (BV/TV), (**e**) trabecular (Tb) number and (**f**) Tb spacing. (**g**) 3D rendering of L3 vertebral bodies from 3 month-old (3 m), and (**h**) 6 month-old (6 m) *WT* vs *Col6α2*-KO mice. (**i**) Quantitative µCT analysis of BMD, (**j**) BV/TV, (**k**) Tb number and (**l**) Tb spacing. 3D Images from each group were obtained from animals with median BV/TV. N = 6/genotype.

To determine how the bones of *Col6a2*-KO mice are affected, isolated femora and vertebrae (L3) were dissected and subjected to μCT analysis (representative 3D constructions shown in Fig. 2a, b, g, h). At 3 months of age, the femora from the *Col6a2*-KO mice had significantly lower BMD (Fig. 2c), bone volume/tissue volume (BV/TV) (Fig. 2d) and Tb number (Fig. 2e), and higher Tb spacing (Fig. 2f) than *WT* mice. These changes persisted with aging and all were still evident in the same parameters when tested in 6 month-old mice (Fig. 1c–f). The L3 vertebra showed a similar pattern of low bone mass phenotype in the *Col6a2*-KO mice with a lower BMD (Fig. 2i), BV/TV (Fig. 2j) and Tb number (Fig. 2k) and higher Tb spacing (Fig. 2l) compared with *WT* mice.

### Cortical bone is not affected in the *Col6a2*-KO mice

**Figure 3:**
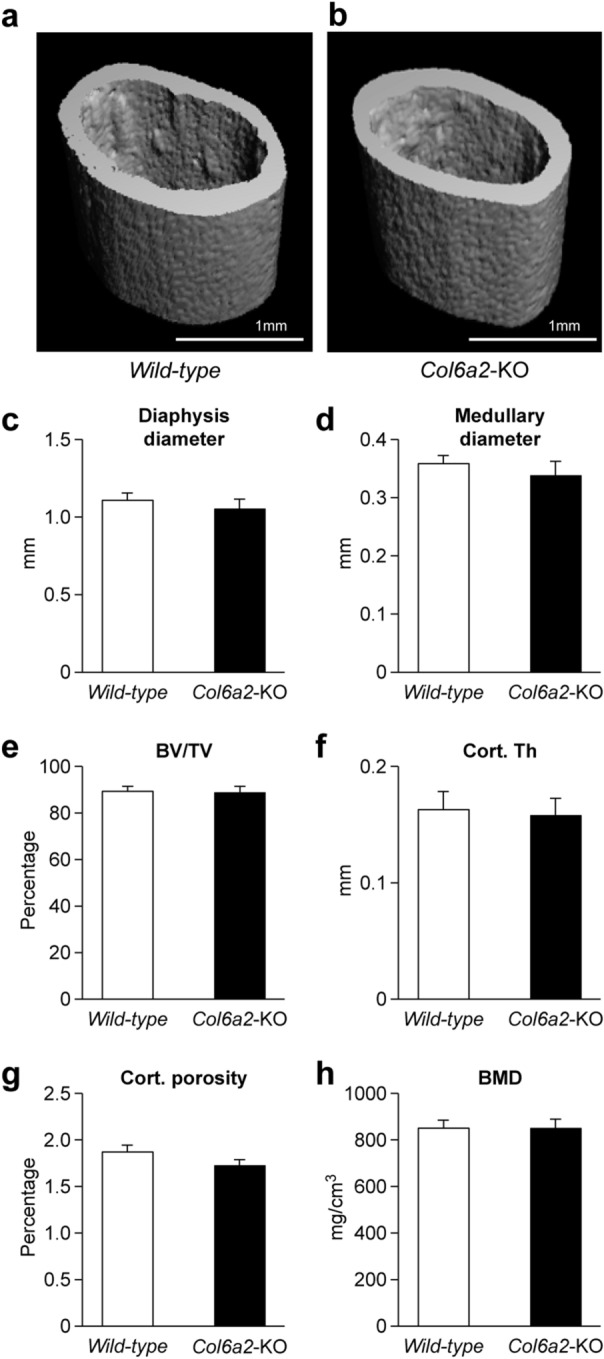
Cortical dimensions of *Col6a2*-KO vs. *WT* mice. 3D µCT reconstruction of femoral mid-diaphyseal cortical bone at 3 months of age from *WT* (**a**) and *Col6a2*-KO (**b**) mice. Images from each group were obtained from animals with medial cortical thickness bone, (**a**, **b**), representative images. Quantitative µCT analysis of (**c**) diaphysis diameter, (**d**) medullary diameter, (**e**) Bone Volume/Total Volume (BV/TV), (**f**) Cortical Thickness (Cort. Th), (**g**) Cortical (Cort.) porosity, and (**h**) Bone Mineral Density (BMD). No significant differences were found in any of the parameters tested (**c**–**h**) between *WT* (**a**) and *Col6a2-*KO mice. N = 8/genotype.

Beyond the trabecular compartment, the mid-diaphyseal cortical femora were assessed for changes associated with the absence of Col6a2. μCT analysis found no striking differences in the appearance of the cortices between genotypes (Fig. 3a, b, left panel *WT*, right panel *Col6a2*-KO). Quantification of the cortical area showed no significant differences in diaphysis diameter (Fig. 3c), medullary diameter (Fig. 3d), BV/TV (Fig. 3e), cortical (Cort.) thickness (Th) (Fig. 3f), Cort. porosity (Fig. 3g) or BMD (Fig. 3h) in the *Col6a2*-KO compared with WT controls.

### *Col6a2-*KO mice have no change in bone formation parameters but have increased osteoclastogenesis

**Figure 4:**
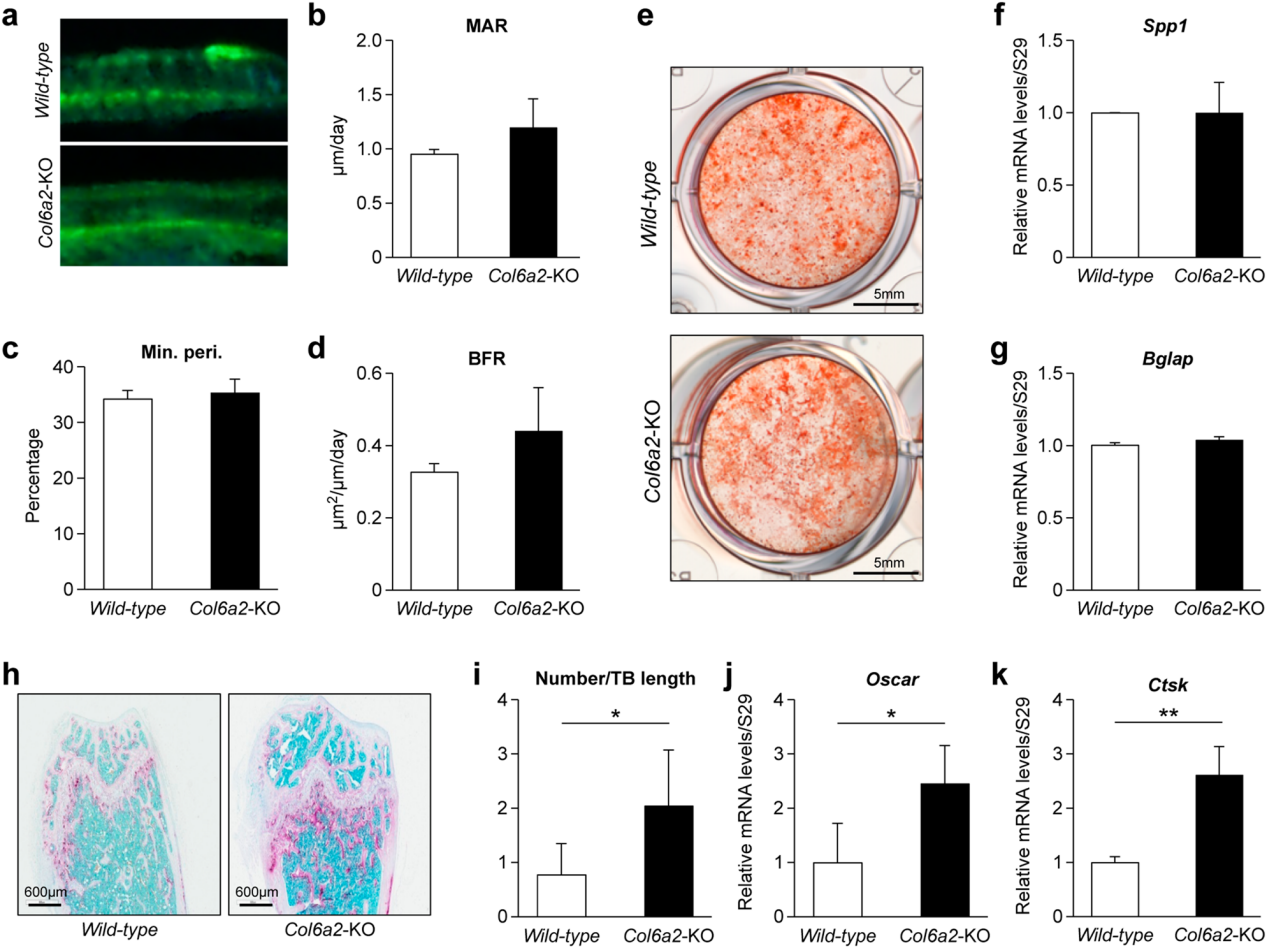
*Col6a2-*KO have increased osteoclastogenesis with no change in bone formation parameters. (**a**–**d**) Dynamic histomorphometric parameters based on fluorescent visualization of calcein fluorochrome in the trabecular compartment of the distal metaphysis of the femur. (**a**) Top panel representative picture from *WT*, bottom panel, from *Col2a2-*KO mice. (**b**) mineral apposition rate (MAR), (**c**) mineralized perimeter (Min. Peri.) and (**d**) bone formation rate (BFR) between wild type and *Col2a2-*KO mice. N = 5/genotype. (**e**) Alizarin red accumulation in BMSCs from wild-type and *Col2a2-*KO mice cultured in osteogenic media, representative image (**f**) Relative mRNA expression of osteopontin (*Spp1*) and (**g**) osteocalcin (*Bglap*). (**h**) Representative images of histological sections of distal femoral metaphysis stained with TRAP from *WT* and *Col2a2-*KO mice, (**i**) quantitation of osteoclast number/trabecular bone length (N/Tb.Le) from *WT* and *Col6a2-*KO, N = 6/genotype. (**j**) Relative mRNA expression of *Oscar* and (**k**) *Ctsk* in *WT* and *Col2a2-*KO derived cells, n = 3 bones/genotype with biological triplicates for each bone. *p < 0.05, **p < 0.01.

To determine the cellular basis for the low bone mass phenotype in the *Col6a2*-KO mice, dynamic histomorphometry was performed using double fluorochrome labeling techniques (Fig. 4a). This analysis demonstrated that there were no significant differences in between Col6a2-KO and *WT* mice in the mineral apposition rate (MAR), (Fig. 4b) or Mineralizing perimeter (Min.Peri.) (Fig. 4c) or bone formation rate (BFR) (Fig. 4d). Alizarin red staining of BMSCs cultured under osteogenic conditions showed no appreciable differences in osteoblast differentiation (Fig. 4e). Relative mRNA expression levels of the osteoblast-expressed genes osteopontin (*Spp1*) (Fig. 4f) and osteocalcin (*Bglap*) (Fig. 4g) were similar in *WT* and *Col6a2*-KO-derived cells. To examine osteoclastogenesis levels, bones were analyzed by TRAP staining. This revealed that the *Col6a2*-KO mice had significantly more positive staining (pink stain) compared with *WT* mice (Fig. 4h). Quantitation of the TRAP stain showed that the number of osteoclast/trabecular length was significantly higher in the *Col6a2*-KO than *WT* mice (Fig. 4i). Relative levels of osteoclast-expressed genes were also higher in the *Col6a2-KO* bones as judged by the expression of Osteoclast-associated Ig-like receptor (*Oscar*) (Fig. 4j) and Cathepsin K (*Cstk*) (Fig. 4k).

### Transcriptome profiling shows dysregulation of osteoclast regulatory pathways in *Col6a2*-KO bones related to bone remodeling

**Figure 5:**
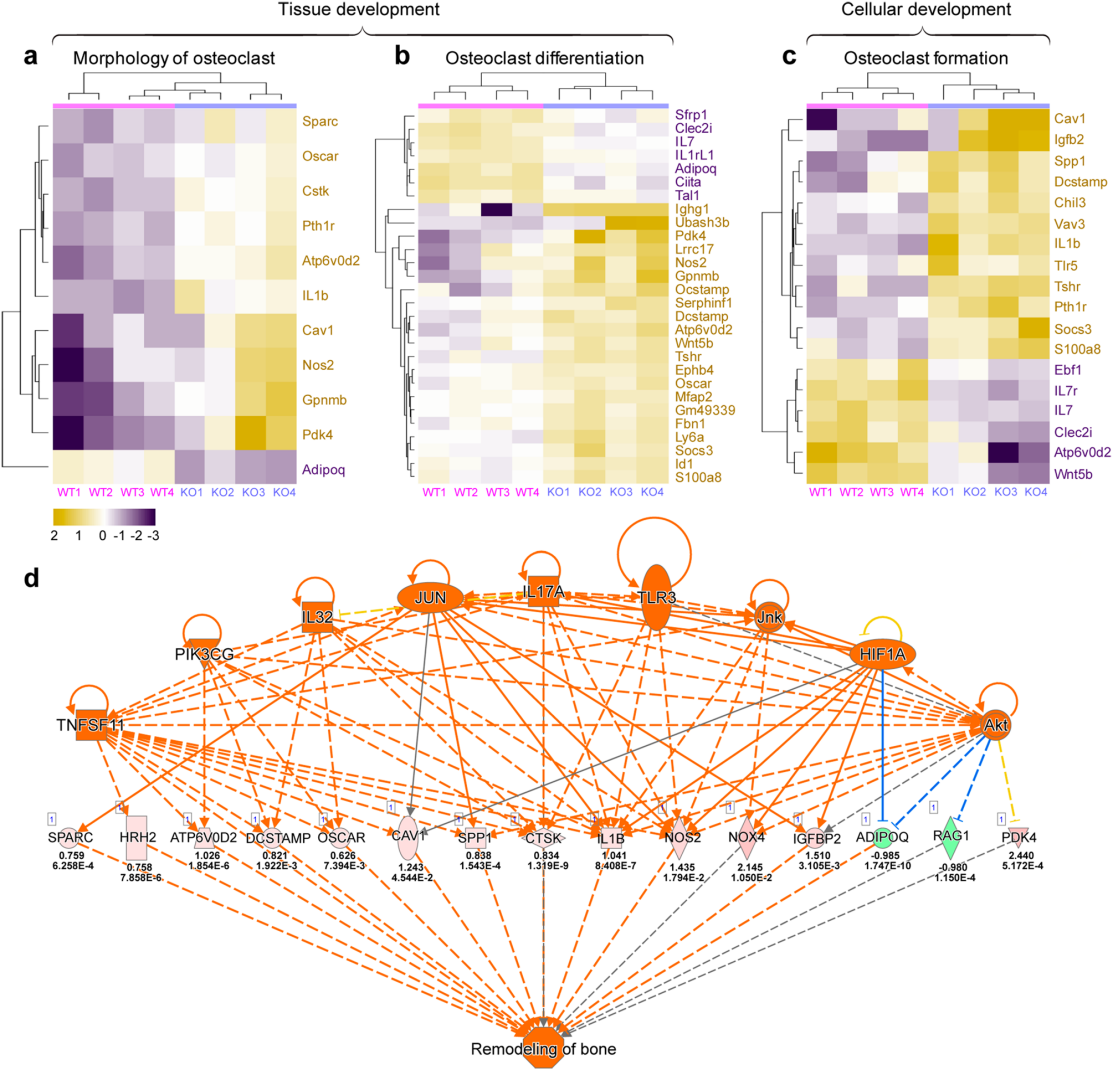
Relative mRNA expression patterns related to osteoclast function between *WT* and Col6a2-KO mice. RNA was extracted from the femora bones from 4 separate wild-type (*WT* 1-4) and *Col6a2-*KO (KO1-4) mice and subjected to RNAseq and differentially expressed genes were further applied to IPA. Heat maps show relative expression levels of genes related to osteoclast (**a**) morphology, (**b**) differentiation and (**c**) formation, with purple blocks being down regulated and yellow being upregulated. Individual genes are shown to the right of the heat maps. (**d**) Interactome analysis of dysregulated genes and pathways in the transcriptome of wild-type and *Col6a2-*KO. The orange ovals/triangle/squares show over 9 different affected regulators (top row) that cascade to influence other sets of genes shown in pink (as up) and green (as down) (middle row). The numbers below the genes show log2 fold change and p-values. Orange colored shapes predict activation. Orange or blue lines lead to activation or inhibition, respectively. This latter set of genes all coalesced and integrated with functions related to bone remodeling.

To broaden our analysis of osteoclast-related genes, RNAseq was performed on bones from 3 month-old W*T* and *Col6a2-*KO mice. Bioinformatic analysis identified a total of 1,107 genes (466 down and 641 upregulated) shown in the heatmap as yellow (up) and purple (down), with clusters linked to the osteoclast morphology (Fig. 5a), osteoclast differentiation (Fig. 5b) or osteoclast formation (Fig. 5c). This included *Oscar* and *Ctsk* that we previously showed to be up-regulated in the *Col6a2*-KO bones using conventional real-time RT-PCR (Fig. 4j,k). Futher analysis of the affected interactomes pointed to numerous key drivers (*Tnfsf11*, *Pik3cg*, *Il32*, *Jun*, *Il17a, Tlr3*, Jnk, *Hif1a*, and *Akt* (Fig. 5d) that act as upstream of effectors of osteoclastogenesis. These effectors influence expression of genes including *Sparc, Hrh2*, *Atp6vd2*, *Dcstamp*, *Oscar*, *Cav1*, *Spp1*, *Ctsk*, I*l1b*, *Nos2*, *Nox4*, *Igfbp2*, *Adipoq*, *Raq1*, and *Pdk4* with pink or green-colored shapes shown as up and down-regulation, respectively, (middle row showing raw data), many of which are connected to bone remodeling. These data support the hypothesis that bones from *Col6a2-*KO mice have overactive osteoclastogenesis and revealed additional molecular players that could potentially contribute to the biological outcome of low bone mass in the *Col6a2*-KO mice.

### Myeloid osteoclast precursor number is not altered in *Col6a2*-KO mice, but response to TNF α is increased in osteoclast precurosors pretreated with BMSC-produced Col6a2

**Figure 6:**
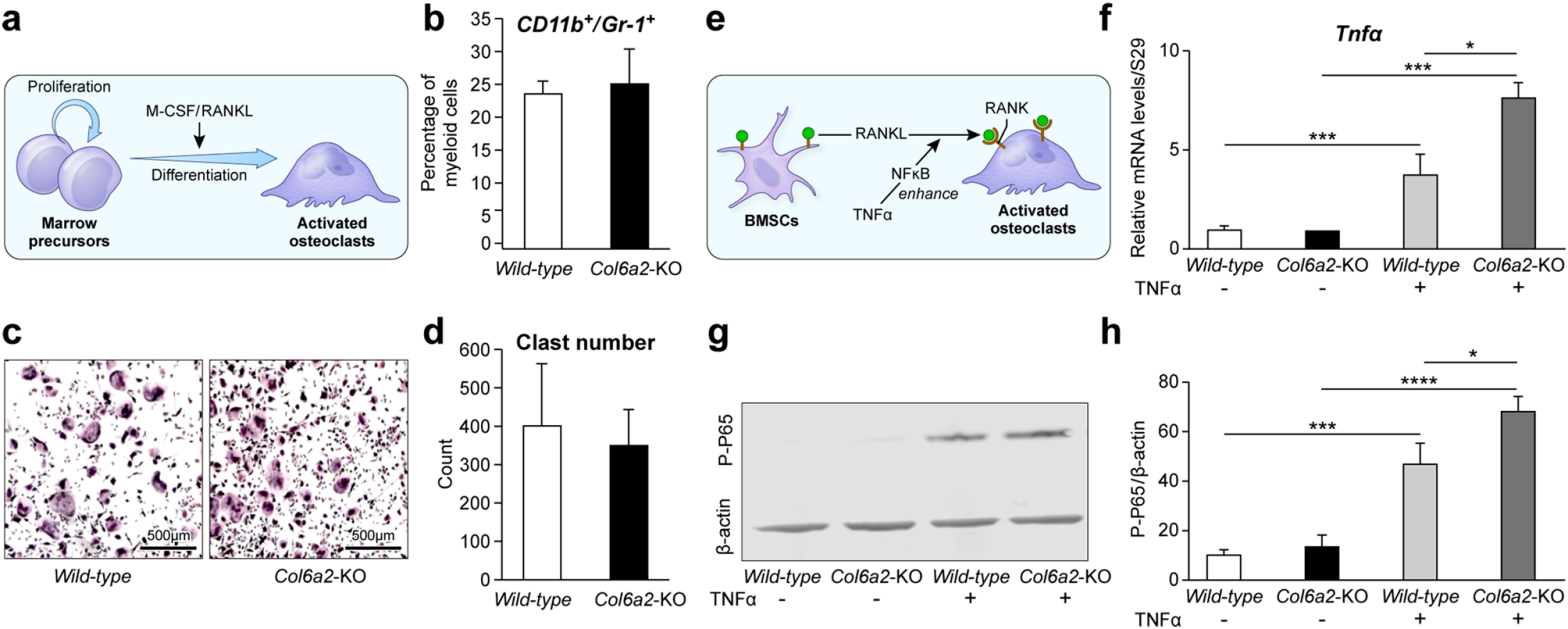
(**a**) Model showing the relationship between bone marrow cell precursors and osteoclasts highlighting the importance of M-CSF/RANKL. (**b**) Percent myeloid cells (osteoclast precursor) judged by CD11b + /Gr-1 + surface markers, N = 3 biological replicates/genotype (**c**) Representative pictures of TRAP staining of cultured osteoclast progenitors treated with M-CSF and RANKL in *WT* compared to *Col6a2*-KO mice. (**d**) Quantitation of osteoclast number showing no differences between *WT* and *Col6a2*-KO mice, N = 4 biological replicates/genotype. (**e**) Model showing potential role of TNFα in the functional coupling of BMSCs and osteoclasts. (**f**) Relative mRNA levels of *Tnfa* in *WT* and *Col6a2*-KO BMSCs treated with or without TNFα, N = 3 biological replicates/genotype with triplicate technical replicates. (**g**) levels of the TNFα target transcription factore p-65 in *WT* vs *Col6a2*-KO mice treated with and without TNFα in the presence of conditioned media from *WT* vs *Col6a2*-KO mice with quantitation, N = 4 biological replicates (**h**) using β-actin as loading control. *p < 0.05, ***p < 0.001, ****p < 0.0001.

Our TRAP staining and RNAseq data pointed towards osteoclast involvement in the low bone mass phenotype of the *Col6a2*-KO mice. To determine if this was due to an increased number of osteoclast precursors (Fig. 6a), the percentage of myeloid cells positive for CD11b and Gr-1 was measured. This analysis showed that there were no significant differences in the percent of CD11b^+^/Gr-1^+^ cells in the *WT* compared with *Col6a2*-KO mice (Fig. 6b). There was also no significant difference in the number of osteoclast precursors differentiated in the presence of M-CSF and RANKL between the *WT* vs *Col6a2*-KO mice (Fig. 6c, d). This indicates that the *Col6a2* deficient osteoclast precursor number and differentiation capacity was not the cause of the low bone mass phenotype. Further analysis of the RNAseq data predicted that TNFα, a factor known to influence osteoclastogenesis was an upstream regulator in the *Col6a2*-KO cells. (Supplemental Table 2), (Fig. 6e). To test the possibility that TNFα activity is altered in the *Col6a2-*KO cells we treated *WT* osteoclast progenitors with or without conditioned media from *WT* or *Col6a2-*KO bone marrow stroma cells (BMSCs) (the source of Col6a2). The Relative mRNA levels of *Tnfa* in *WT* and *Col6a2*-KO BMSCs treated with or without TNFα showed that the response to TNFα was more effective in BMSCs treated with conditioned medium from *Col6a2*-KO cells compared with conditioned medium from *WT* BMSCs (Fig. 6f). The phosphorylated form of p65, which is a down-stream effector of TNFα, was also measured. In response to TNFα, BMSCs treated with *Col6a2*-KO BMSC-conditioned medium induced more phosphorylated p65 than BMSCs treated with *WT* conditioned medium (Fig. 6g, h). These findings all point to a role for TNFα in the overactive osteoclastogenesis observed in the *Col6a2*-KO mice.

### Col6a2 directly binds to TNF-α in vitro and can reduce osteoclastogenesis in TNFα-treated osteoclast precursors

**Figure 7:**
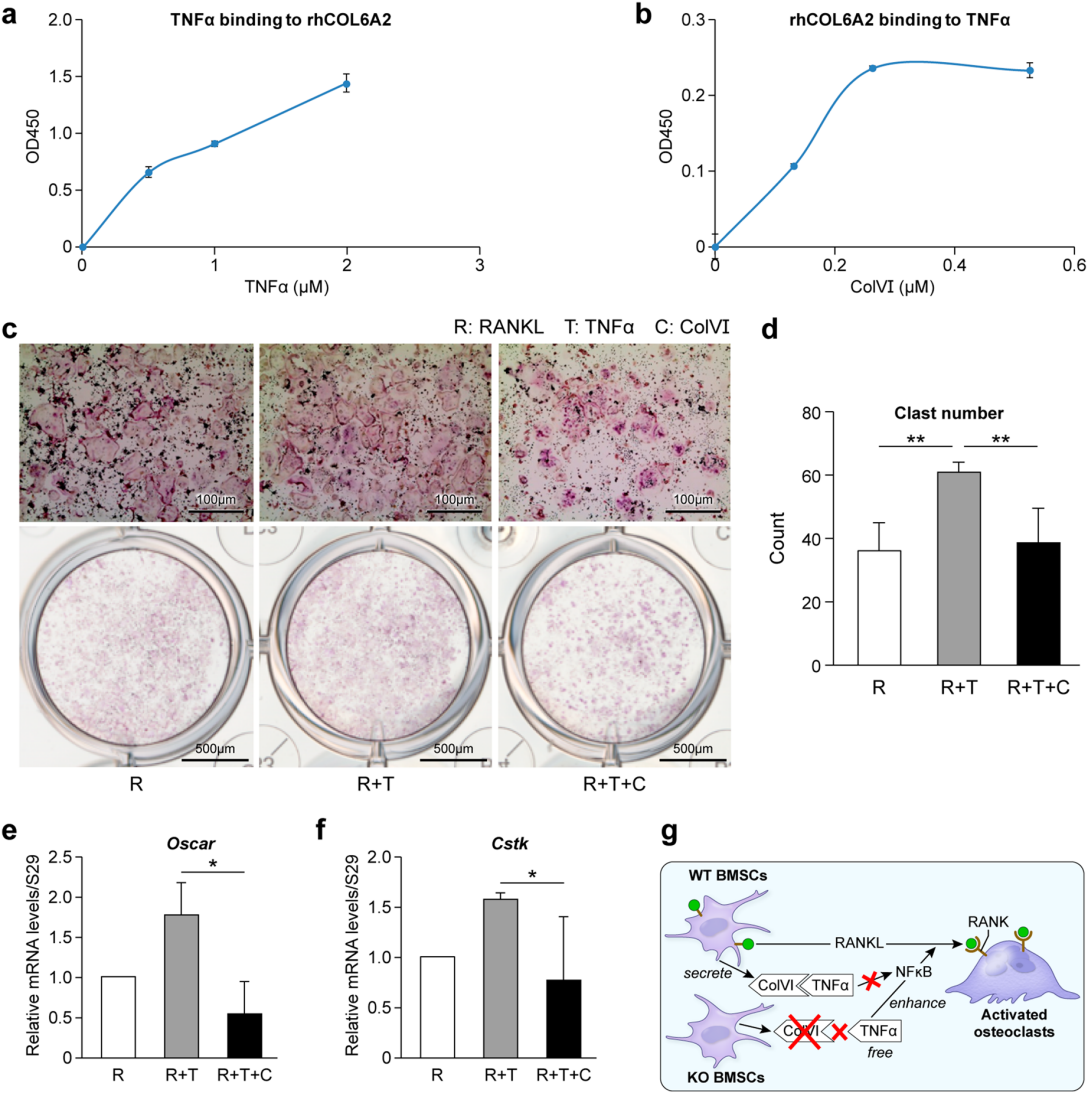
ColVI binds to TNFa and reduces TNFa induced osteoclastogeneis. (**a**, **b**) solid-phase binding assay. (**a**) rhCOl6A2 was bound to plates and treated with increasing concentrations of TNFα. (**b**) TNFα was bound to plates and treated with increasing concentrations of rhCOl6A2, representative graphs, Data are mean ± SE obtained from N = 3 independent experiments. (**c**–**g**) Inhibition assay. (**c**) TRAP staining of cultured RAW 264.7 cells treated with RANKL, TNFα or ColVI and (**d**) quantitative number of osteoclasts in those cultures, (**f**, **g**) relative mRNA levels of osteoclast markers *Oscar* and *Cstk* , N = 4 independent experiments. (**h**) Diagram showing the proposed inhibitory function of ColVIα2 to reduce the effects of TNFα on osteoclastogenesis. The scheme proposes that ColVI made by BMSCs binds to extracellular TNFα and reduces its ability to stimulate osteoclastogenesis. *p < 0.05, **p < 0.01.

To determine how Col6a2 could potentially regulate TNFα activity, we tested the possibility that Col6a2 could directly bind to TNFα and thereby harness its activity. We coated plates with a recombinant human (rh) COL6A2 fragment (designated rhCOL6A2) and added increasing amounts of TNFα. We found a dose-dependent binding of TNFα to the rhCOL6A2-coated plates (Fig. 7a). When the experiment was done using TNF-α coated plates we again saw a dose response in binding of rhCOL6A2 (Fig. 7b). Next, we treated the osteoclast precursor RAW 264.6 cell-line with either RANKL (to stimulate osteoclastogenesis), and either TNFα alone (to further enhance osteoclastogenesis) or with ColVI (total human protein), which we predicted would dampen osteoclastogenesis. Our data showed that the presence of TNFα could stimulate RANKL induced osteoclastogenesis (Fig. 7c), and that adding ColVI to the RANKL and TNFα-induced cultures reduced osteoclast number (Fig. 7d), and differentiation judged by the expression of *Oscar* (Fig. 7e), and *Cstk* (Fig. 7f). Taken together, these data suggest that ColVI produced by BMSCs binds to TNFα and reduces its ability to stimulate osteoclastogenesis (Fig. 7g). When ColVIa2 is depleted (causing a reduction in total COLVI, see Fig. 1), TNFα is not sequestered in the extracellular matrix and is free to enhance the actions of RANKL on osteoclastogenesis (Fig. 7g).

## Discussion

It has been known for some time that the extracellular matrix (ECM) plays an essential role in mineralized tissue and yet its exact roles in regulating bone homeostasis are not fully understood^9^. Several “key players” have been identified that are embedded in the mineral compartment of bone that include type I collagen and non-collagenous proteins such as proteoglycans^10,11^. Considering the uniqueness of hard tissue, many have searched for a component in the ECM that would control bone mass accrual and subsequent biomineralization. While many factors may contribute to bone formation and bone turnover, their precise nature and interaction with other systemic or local factors is in need of clarification. In an attempt to deepen our understanding of ECM components in bone health, we have focused on ColVI, which we and others have found to be abundant in forming bone.

By creating mice globally deficient in *Col6α1,* Cescon et al. described a high versatility for this protein, showing its involvement in many tissues, including muscle, skin, adipose tissue and the nervous system^5^. Its role varies depending on tissue context, and affects a wide range of processes including apoptosis, tumor growth, and autophagy^5^. In the nervous system, ColVI is needed for peripheral nerve regeneration in a process dependent on macrophage recruitment and polarization^12^.

ColVI is highly expressed in the osteogenic lineage^13^ suggesting that it has a function in bone. When the *Col6α*1KO mice were examined for bone parameters it was found there was reduced trabecular bone volume starting at 2 months that persisted until 9 months and leveled off by 15 months of age^14^. The reduced trabecular bone in the *Col6α1-*KO mice is accompanied by an increase in trabecular structure model index (SMI) at 2, 9 and also at 15-months of age compared with *WT* mice, suggesting that ColVI has a significant role in regulating normal bone homeostasis. Other studies using the same *Col6a1-*KO mouse model confirm its importance in maintaining bone mass and suggest this could arise from defects in osteoblasts located in the periosteum, the thin layer of tissue that surrounds the bone. The osteoblasts in the *Col6a1-*KO are less cuboidal or “plump” and appear more disorganized than WT counterparts^15^. Mice deficient in ColXII (*Col12a1KO)* also show a similar osteoblast disorganization, leading to the speculation that ColVI and ColXII could interact and somehow regulate osteoblast integrity^16^. In an in vitro osteoblast differentiation system, Izu et al., found that ColVI and ColXII co-localize in bridge-like structures that are thought to be involved in osteoblast communication. Interestingly, the matrix-rich bridge structures containing ColVI or ColXII were reduced in both the *Col6α1-*KO and the *Col12a1-*KO, suggesting they are functionally dependent on each other for network and bridge assembly during bone formation^16^. What is not known from these studies is how bone turnover could be affected by either ColVI or ColXII. Our work builds and expands upon these investigations and shows, for the first time, a novel role for ColVI in attenuating osteoclastogenesis and, subsequently, bone mass status.

Despite our evidence showing that Col6a2-KO mice have higher levels of osteoclasts than WT mice as 3 months of age, it must be noted that the increase in osteoclast number found in the Col6a2-KO mice did not perfectly match the extent of bone loss found compared with WT mice and, furthermore, was not progressive with age as would be expected. It is possible that even with the apparent increase in osteoclast numbers found that they were not active and did not lead to increased resporption. However, further experiments will be required, such as measurements of biochemical markers of bone turnover in the serum to fully address this question. In addition to this, we find from IHC analysis of bones from 1 day, 1 month and 3 month-old mice that ColVI was expressed at a higher level in young versus old animals (data not shown). Thus, the loss of Col6a2 from birth on that occurs in our mouse model could be affecting trabecular bone formation prior to the time point selected to measure the Mineral Apposition Rate. It is also possible that the expression of ColVI in the growth plate during development affects bone structure and integrity. Additional experiments will need to be needed to fully address these questions.

While it is clear that ColVI potentially has multiple functions throughout the body, its role in muscle is the most thoroughly studied and could help inform us about its roles in the skeletal system. Numerous human mutations in the ColVI genes occurring in all three alpha chains, lead to either Bethlem myopathy, at the mild end of the disease spectrum, or Ullrich congenital muscular dystrophy, a severe debilitating disorder^17^. Insterstitial fibroblasts are the main source of ColVI in muscle tissue and efficient secretion from the cell is needed for its proper function^18^. Currently, very little is known about how mutations in human ColVI genes affect bone function. A previous study designed to test the effects of cyclosporin in ameliorating the muscular symptoms of UCMD used whole body DEXA scans, and their analysis showed no significant differences in BMI, Lean Tissue Mass, Fat Tissue Mass, %Fat or BMD before and after cyclosporin treatment^19^. Despite the apparent negative outcome of the investigation, the patients with mutations in either COL6A1, COL6A2 or COL6A3 all had BMD Z scores significantly below the norm. These data imply a role for ColVI in human bone health^19^. COLVI levels decrease with age in human osteoporotic bone^20^, further pointing to a possible role for COLVI in bone homeostasis with aging. Reduced BMD in patients with COLVI mutations could result from the influence of the defective muscle on bone tissue. Decreased muscle activity could reduce the biomechanical forces on bone and lead to reduced BMD, or alternatively, patients could have defective cytokine interplay between muscle and bone. The use of animal models with tissue-specific ColVI (bone vs. muscle), or other experiments using co-cultures or conditioned media from muscle- and bone-derived cells^21^ could help resolve this important question.

The immune and skeletal systems have a close association, where immune cells mediate powerful effects on bone turnover^22^. It is well-known that chronic inflammation exacerbates bone loss. Tumor necrosis factor alpha (TNFα), a proinflammatory agent, is often involved in inflammatory reactions^23,24^. TNFα is produced as a transmembrane protein (pro-TNFα) that is subsequently cleaved by the TNFα converting enzyme (TACE) to a soluble form^25^. Both forms of TNFα are biologically active, though the membrane-bound form is the one used under normal conditions, while the soluble form is associated with pathology^26–29^. Both forms bind as a trimer to either TNFR1 (also known as TNFRSF1A or p55) or to TNFR2 (also known as TNFRSF1B or p75). Compared with the ubiquitously expressed TNFR1, which has a “death domain” to initiate apoptotic signaling, the expression of TNFR2 is limited to cells of the hematopoietic lineage.

Numerous investigations have examined the relationship between TNFα and osteoclastogenesis. These studies show that even though TNFα on its own does not effectively induce osteoclast differentiation^30–33^, it plays a major role in promoting bone resorption, usually in synergy with RANKL at the signal transduction level^30,34,35^. TNFα indirectly increases osteoclastogenesis through augmentation of M-CSF and RANKL expression in stromal cells while down-regulating osteoblastic production of OPG^36^. It also directly promotes RANK expression on monocytes, thus converting them into osteoclast precursors^37–39^.

TNFα also enhances proteoglycan expression, facilitating ECM remodeling during the early stages of the inflammation process^40^. Moreover, BMSC-secreted SLRPs have been shown to regulate osteoclastogenesis via their interaction with TNFα^41^. It is likely that a similar mechanism is used by ColVI produced by BMSCs to attenuate osteoclastogenesis. TNFα inhibits nitric oxide (NO) production and intracellular calcium, while strongly reducing F-actin content in osteocytes, leading to a reduction in osteocyte stiffness, which in turn induces their apoptosis^42^. Interestingly, apoptotic osteocytes are known to attract osteoclasts, pointing to the possibility that osteocytes are also affected in the *Col6a2*-KO mice, but this is yet to be determined.

Despite our finding that ColVI binds to TNFα and inhibits osteoclastogenesis in vitro*,* we have not yet provided direct proof that this occurs in vivo*.* In this regard, further experiments will be needed using inhibitors to TNFα or other genetically altered mice to prove that the association of TNFα with ColVI is valid and has an impact in the context of an in vivo situation. We are also not sure why there were no significant effects of Col6a2 loss on cortical bone but suggest that one reason could be that there are many more osteoclasts in the trabecular bone compared with cortical bone, at least at the 3-month time point analyzed. Still another possibility is that trabecular bone is much more metabolically active than cortical bone and is “primed” for environmental cues such as changes in TNFα activity. It could also be that there are other factors working in conjunction with Col6a2 that are more affected in the trabecular versus the cortical compartment, but a true understanding of differential effects on different bone regions will only come with further investigation.

ColVI binds to a wide range of the ECM components besides TNFα. The proteoglycan, NG2, is needed to retain ColVI at the cell membrane^43^ by ColVI binding to the central nonglobular domain of the NG2 core protein^44^. NG2 expression is reduced in skeletal muscle of UCMD patients suggesting it has some connection to this muscle pathology^45^. NG2 is also found in growing bone and colocalizes with ColI in the ECM of the osteonal Haversian canal^46^. While the role of NG2 in the osteon is not definitively understood, its location coincides with Runx2-positive and PCNA-positive cells suggesting that the combination of ColVI and NG2 could provide an extracellular microenvironment conducive for proliferation and differentiation of osteoblastic lineage cells^46^.

In addition to NG2, ColVI also binds to matrilin-1, decorin and biglycan^47^. Biglycan is a SLRP already known to be important in bone^48,49^ that co-localizes to similar regions as ColVI in bone, making it an attractive candidate for further investigation in this context.

When the biglycan-ColVI interaction was tested using fragments or isolated ColVI chains, the strongest binding was with the Col6a2 chain, revealing its importance in this matrix–matrix interaction^50^. The biglycan-ColVI interaction leads to the formation of hexagonal-like networks that resemble tissue structures when visualized by electron microscopy (EM)^51^. Comprehensive genotype–phenotype analysis of patients harboring mutations in COL6A1, COL6A2 or COL6A3 shows a cluster of mutations in the N-terminal region of the triple helix that produce severe phenotypes, indicating this could be a functional domain in the triple helix^52^. Indeed, previous EM investigations show that both biglycan and decorin bind to a domain in ColVI that is close to the interface between the N terminus of the triple helical region and the neighboring globular domain^50^. This observation further supports the concept that the N-terminus of COLVI has key roles in its function. Unclear at this time is how COLVI interactions with biglycan, decorin or even matrilin-1 might regulate its functions in bone.

In summary, we show for the first time, a role for Col6a2 in regulating bone mass. We further show Col6a2 regulates bone loss by modulating TNFα-induced osteoclastogenesis. Exactly which region of Col6a2 controls this function is still unknown, as well as the possible interplay with additional binding partners such as biglycan. It will also be interesting to determine if Col6a2 plays a role in bone healing, a process that is distinct from normal bone turnover shown in the present investigation.

## Materials and methods

### Animal experiments

The *Col6a2*-KOmouse strain used for this research project was created from ES cell clone 12228C-E10, generated by Regeneron Pharmaceuticals, Inc. in the KOMP Repository (https://www.komp.org) and the Mouse Biology Program (https://www.mousebiology.org) at the University of California-Davis, and then made into live mice that were backcrossed to the C57B6J strain for 5 generations. Animals were housed under standard conditions (55% humidity, 12 h day night cycle, standard chow and free access to water) following the guidelines and approval of The National Institutes of Dental and Craniofacial Research Animal Care and Use Committee (protocol #18-865).

#### Bone marrow stromal cell culture (BMSCs)

Mouse BMSCs from both *WT* and *Col6a2-*KO 12-week-old male mice were isolated from the femora and tibiae by flushing the bone marrow and cultured in vitro using α-MEM (Gibco, USA) containing 20% fetal bovine serum (FBS) (Atlanta Biologicals, USA), 1% antibiotics (penicillin 100 units/ml and streptomycin 100 mg/ml), 1% GlutaMax-I (Gibco, USA), and 55 μM β-mercaptoethanol (Life Technologies, NY, USA). After cells reached 70% confluence (passage 0) they were trypsinized and suspended for use in the cell culture assay.

#### Osteoclast cultures

##### Primary osteoclast culture

 Mouse bone marrow cells from 12-week-old male *WT* and *Col6α2* KO mice were isolated from femora and tibiae by flushing the bone marrow with 25- and 27-gauge needles, respectively, and filtered with a 70-μm cell strainer (Falcon, USA). Cells were centrifuged at 1,600 rpm for 10 min and re-suspended in α-MEM medium containing 10% FBS, 1% penicillin 50 units/ml, and 1% fungizone 1.25 μg/ml. Cells were seeded in 10 ml/10 cm^2^ culture dish. After 3 h, the supernatant was collected and seeded into a new 10-cm^2^ culture dish for 24 h. The supernatant was re-collected and centrifuged at 1,400 rpm for 5 min. The pellets were re-suspended for use in the osteoclastogenesis assay. The cells were plated at 500,000 cells/cm^2^ in a 96-well plate with 40 ng/ml of RANKL (R&D, USA) and 30 ng/ml of M-CSF (R&D, USA) to induce osteoclastogenesis. The induction medium was changed every other day, samples were harvested at day 5 and fixed and stained with a TRAP staining kit (Sigma, US) following the manufacturer’s instruction. The images were observed under an EVOS XL Core microscope (Thermo Fisher, USA) and analyzed by Image Pro 7.0 software (Media Cybernetics, USA).

##### RAW 264.7 cell culture

The RAW 264.7 cell line was purchased from ATTC (TIB-7) and cultured in DMEM (ATCC) containing 10% FBS, 1% penicillin 50 units/ml. To perform the osteoclast assay, 20,000 cells were plated in 96 wells plate with 30 ng/ml of RANKL (R&D, USA) and simultaneously treated with or without 10 ng/ml of TNFα (R&D, USA), and 100 ng/ml of human COLVI (SouthernBiotech, USA). The medium with factors was replenished every other day, samples were fixed at day 5, and stained and imaged as described above.

#### Dual-energy X-ray absorptiometry (DEXA)

To determine the bone mineral density, whole mouse bodies (the skull were excluded from region of interest when analyzing) and femurs were scanned with a DEXA machine (Lunar PIXImus densitometer, GE Healthcare) and Faxitron Ultrafocus (Faxitron, USA), respectively.

#### Micro-computed tomography (μCT)

Right femurs and third lumbar vertebrae (L3) from 3 and 6 month-old *WT* and *Col6a2* KO mice were surgically collected and fixed for 24 h at room temperature in Z-fix (170; Anatech, LTD), then stored in 70% ethanol at 4 ºC. Samples were scanned by µCT (µCT 50, Scanco Medical AG, Bassersdorf, Switzerland) at a resolution of 10 μm and reconstructed using the global approach with energy at 70 kV, intensity/beam current at 85uA, power at 6 W and Integration time 300 ms^53^. Trabecular and cortical bone was quantified according to previously published guidelines^54^. For trabecular bone, the following parameters were analyzed: bone volume/tissue volume (BV/TV), trabecular thickness (Tb.Th), trabecular number (Tb.N), and trabecular spacing (Tb.S). For cortical bone, (BV/TV), cortical thickness (Cort.th), cortical porosity (Ct. Po), bone mineral density (BMD), diaphysis diameter (Dp.dm) and medullary diameter (Me.DM) were assessed. All cortical bone measurements were on bones from 3 month-old mice.

#### TRAP analysis

Femurs were fixed in Z-fix for 24 h, rinsed with PBS overnight and decalcified with 10% EDTA for 5 days. Samples were then washed and dehydrated through a graded ethanol series and xylene before paraffin embedding. The blocks were sectioned at 5 μm thickness, deparaffinized, stained with H&E or performed with tartrate resistant acid phosphatase activity (TRAP) (Wako Pure Chemical Industries, Ltd., Osaka, Japan), and observed under an Aperio ScanScope (Leica ICC50 W, Germany).

#### Immunofluorescence staining

For preparation of frozen sections from non-fixed and non-decalcified hard tissues, Kawamoto’s film methods were used. Briefly, samples were freeze-embedded with super cryoembedding medium (SECTION-LAB Co. Ltd., Hiroshima, Japan), and 3 um sections were cut using a tungsten carbide blade after attaching the adhesive film onto the sample surface. Samples were then immediately fixed with 4% paraformaldehyde (PFA) for 5 min. For analysis of ColVI, its primary antibody (70R-CR009x, Fitzgerald, USA) was incubated at 4 °C overnight, after blocking with 10% normal donkey serum (Jackson Immunoresearch, USA) for 60 min at room temperature. Primary antibody was diluted to 1:50. After washing, the specimens were incubated with secondary antibody, Alexa Fluor 647 donkey anti-rabbit IgG (Jackson Immunoresearch, USA) for 60 min at room temperature. All images were taken by fluorescence microscope.

#### Dynamic histomorphometry

For in vivo calcein double labeling, 12-week old mice were injected intraperitoneally with a 15 mg/kg of calcein fluorochrome (Sigma-Aldrich, St. Louis, MO) at 7 days and 1 day prior to euthanasia. The femora were collected and embedded undecalcified in methyl methacrylate, then coronally sectioned. Mid-frontal sections were scanned with a fluorescent microscope (Olympus DP72, Japan) and analyzed by Image Pro 7.0 software (Media Cybernetics, USA). For in-vivo osteoclast assessment, paraffin-embedded femoral histological sections were stained with a tartrate-resistant acid phosphatase (TRAP) staining kit (Wako, Osaka, Japan), following the manufacturer’s protocol, and observed using an Aperio ScanScopes (Leica ICC50 W, Germany). The number of osteoclasts was counted by Image Pro 7.0 software (Media Cybernetics, USA).

#### mRNA extraction and real time reverse-transcription polymerase chain reaction (real time RT-PCR)

2-month old WT mouse femora were isolated, immediately frozen in liquid nitrogen, and stored at -t80 ºC. They were then put into the center of a tissue tube (Covaris, USA), frozen in liquid nitrogen, and pulverized on the CP02 cryoPREP Automated Dry Pulverizer (Covaris, USA) to disrupt the extracellular matrix. Total tissue RNA was extracted by TriPure (Sigma, USA) /RNeasy (Qiagen, Germany) hybrid extraction protocol. Total RNA was extracted from cell cultures using RLT buffer (Qiagen, Germany) and isolated using the TriPure/RNAeasy system described above. cDNA was synthesized with an iScript cDNA Synthesis Kit (Bio-Rad, USA). For both in-vivo and in-vitro samples, RT-PCR was performed with primers, iQ SYBR (Bio-Rad, USA) and a CFX96 Real-Time PCR detection system (Bio-Rad, USA). The relative levels of mRNA of target genes were normalized to the housekeeping gene, S29. Primer sequences are listed in Supplementary Table 1. All experiments employed at least three independent experiments with greater than 3 biological replicates in each experiment.

#### RNA-sequencing and analysis

Total RNA was extracted from 3 month-old mouse femora as described above. Sequencing libraries were prepared using a Nextera XT kit (Illumina), individually barcoded, pooled to a 2 nM final concentration, and sequenced on a NextSeq500 (Illumina) using 75 × 75 single-end reads. After sequencing, the base-called demultiplexed (fastq) read qualities were determined using FastQC (v0.11.2) (https://www.bioinformatics.babraham.ac.uk/projects/fastqc/), aligned to the GENCODE M11 mouse genome (GRCm38.p4) and gene counts generated using STAR (v2.5.2a)^55^. Post-alignment qualities were generated with QoRTS (v 1.1.6) ^56^. An expression matrix of raw gene counts was generated using R (https://www.R-project.org) and filtered to remove low count genes (defined as those with less that 5 reads in at least one sample). The filtered expression matrix was used to generate a list of differentially expressed genes between the sample groups using three statistical methods: DESeq2^57^, EdgeR^58,59^, and Limma-voom^60^. Differentially expressed genes (*Col6a2*-KO vs. *WT*, FC > 1.5, FDR < 0.05) were considered for further analyses based on results from DESeq2. A total of 1,107 genes (466 down, 641 up) were applied to Ingenuity Pathway Analysis (IPA) (Qiagen). IPA-identified candidate genes related to biofunction and regulator effects. Heat maps were generated using shinyheatmap from the Genomics and Computational Biology Core (https://brics.nidcr.nih.gov).

#### Western blotting of treated cell lysates

BMSCs harvested from *WT* and *Col6a2*-KO mice were cultured in basal medium until 60% confluency. Subsequently, the basal medium was changed to serum-free medium, then BMSCs were cultured for 2 days to collect conditioned medium containing Col6 secreted by BMSCs. To examine the possible interaction of Col6VI and TNFα, BMSCs were treated with conditioned medium that was supplemented with 10 ng/mL TNFα (R&D systems, Minneapolis, MN) for 15 min. Treated BMSCs were lysed using M-PER (Thermo, Waltham, MA), supplemented with a Protease Inhibitor Cocktail (Roche, Indianapolis, IN) and PhoSTOP (Sigma-Aldrich, Saint-Louis, MO). Cell debris were removed from lysates by centrifugation at 12,000 rpm for 10 min at 4 °C. The protein concentration in the cell lysate was determined by using the Pierce BCA Protein assay kit (Thermo). Twenty micrograms of the total protein was separated in precast polyacrylamide gels (NuPage; Life Technologies, Carlsbad, CA) by electrophoresis and then transferred onto nitrocellulose membranes at 30 V for 2 h. Blots were blocked and incubated with primary antibodies against phospho-p65 (1:1,000 dilution) or β-actin (8H10D10; 1:5,000 dilution, Cell Signaling Technology, Danvers, MA), which was used as the loading control. Blots were then washed and incubated with IRDye 800CW Goat anti-Rabbit IgG Secondary Antibody (1:5,000 dilution; LI-COR Biosciences, Lincoln, NE) or IRDye 680LT Goat anti-Mouse IgG Secondary Antibody (1:5,000 dilution; LI-COR Biosciences) for 1 h at room temperature. The blots were visualized with a LI-COR imaging system and densiometric analysis done using Image Studio Lite software (LI-COR Biosciences).

#### Solid-phase binding assay

Recombinant human COL6A2 (abcam 169,887) or human TNFα (R&D) proteins were pre-tested to determine the optimal concentration for coating plates used in the binding assay, which was 1 µM and 0.26 µM respectively. Bait proteins were bound to Nickel coated plates (15,242; Thermo scientific) with 1-h incubation at room temperature. Unbound proteins were washed off and non-specific binding sites was blocked with 1%BSA/TBS. hCCOL6A2 protein-coated plates were incubated with different concentrations of prey proteins, mTNFα (R&D). In the reverse experiment hTNFα coated plates were incubated with different concentration of hCOL6A2 at 4 °C overnight. Unbound proteins were washed off and binding of the prey proteins to the coated plate was detected using primary antibodies raised against abTNFα (abcam 9,739) or abColVI (70R-CR009X, Fitzgerald), combined with species-matched HRP-conjugated secondary antibodies. Plates were read at 450 nm after HRP chromogenic substrate reaction using the TMB-peroxidase substrate system (KPL, Gaithersburg, MD, USA) according to the manufacturer’s protocol.

#### Statistics

Statistical analyses were performed with unpaired Student`s *t*-test. A statistically significant difference was considered as *p* < 0.05.

#### Study approval

All animal studies were performed in accordance with NIH guidelines under institutionally approved protocols.

## Supplementary information

Supplementary information 1

Supplementary information 2

Supplementary information 3

Supplementary information 4
