# Supplementary material for: Collagen VIα2 chain deficiency causes trabecular bone loss by potentially promoting osteoclast differentiation through enhanced TNFα signaling
Source: Sci Rep. 2020 Aug 13;10:13749. doi: 10.1038/s41598-020-70730-7 (PMC7426410; doi:10.1038/s41598-020-70730-7)
Supplement: Supplementary file 1 — Supplementary information 1 [file 41598_2020_70730_MOESM1_ESM.pptx]

## Slide 1
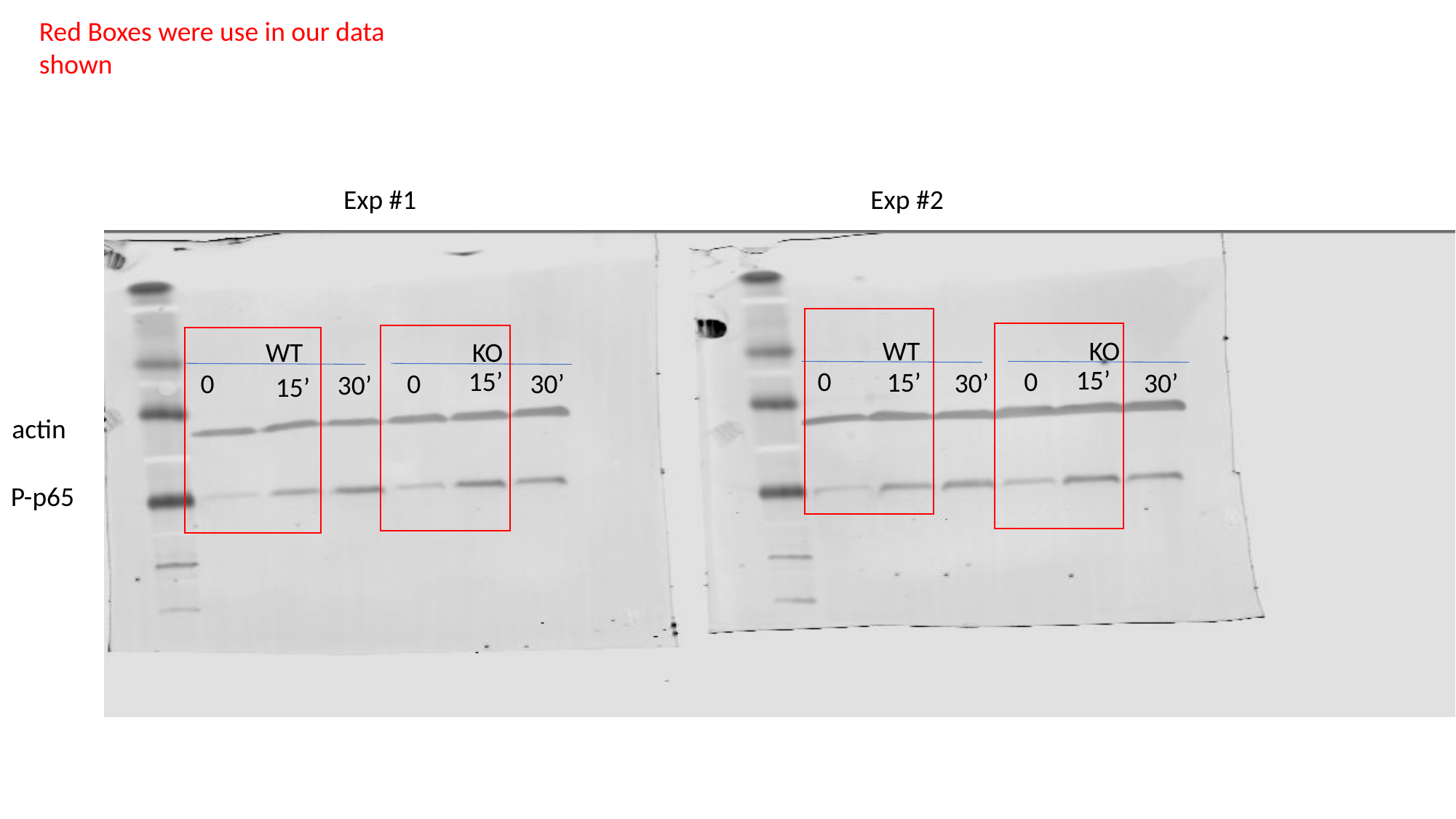

Red Boxes were use in our data shown
Exp #1
Exp #2
WT
0
15’
30’
KO
15’
0
30’
WT
0
30’
15’
KO
15’
30’
0
actin
P-p65

## Slide 2
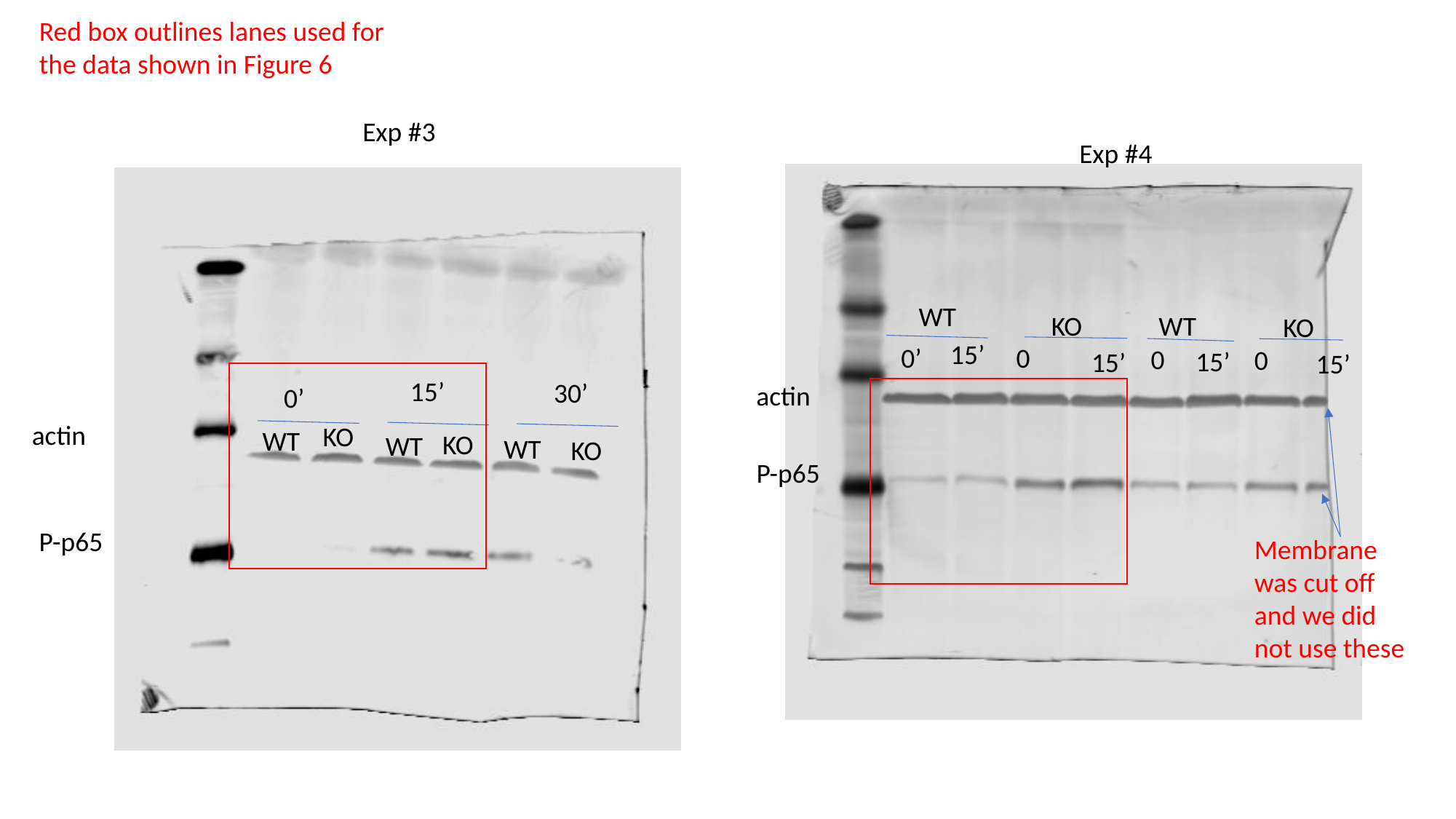

Red box outlines lanes used for the data shown in Figure 6
Exp #3
Exp #4
WT
15’
0’
WT
0
15’
KO
0
15’
KO
0
15’
15’
0’
WT
30’
actin
actin
KO
KO
WT
WT
KO
P-p65
P-p65
Membrane was cut off and we did not use these
