# Supplementary material for: Collagen VIα2 chain deficiency causes trabecular bone loss by potentially promoting osteoclast differentiation through enhanced TNFα signaling
Source: Sci Rep. 2020 Aug 13;10:13749. doi: 10.1038/s41598-020-70730-7 (PMC7426410; doi:10.1038/s41598-020-70730-7)
Supplement: Supplementary file 2 — Supplementary information 2 [file 41598_2020_70730_MOESM2_ESM.docx]

**Supplemental Information File for:**

**Collagen VIα2 chain deficiency causes trabecular bone loss by potentially promoting osteoclast differentiation through enhanced TNFα signaling**

Hai T. Pham, Vardit Kram, Qurratul-Ain Dar, Taishi Komori, Youngmi Ji, Payam Mohassel, Jachinta Rooney, Li Li, Tina M. Kilts, Carsten Bönnemann, Shireen Lamandé

& Marian F. Young^*^

*****To whom all correspondence should be addressed

**Table 1**. List of primers used for Real-Time RT-PCR. F: forward/sense; R: reverse/antisense

**Table 2.** Top 5 upstream regulators. Differentially expressed genes (total 1107, Down: 466, Up: 641) from DESeq2 were applied to Ingenuity pathway analysis (IPA) (Qiagen). IPA identified the top 5 upstream regulators including TNF α.

**Original Western Blot:** Data shown Figure 6G was derived from three separate biological samples labeled Exp #1, Exp #2, Exp #3. Time of the TNF α treatment indicated on the top of the gel and the antibodies used shown on the left side of the gel. KO is Col6a2 and WT the control cells.
