# Supplementary material for: Collagen VIα2 chain deficiency causes trabecular bone loss by potentially promoting osteoclast differentiation through enhanced TNFα signaling
Source: Sci Rep. 2020 Aug 13;10:13749. doi: 10.1038/s41598-020-70730-7 (PMC7426410; doi:10.1038/s41598-020-70730-7)
Supplement: Supplementary file 3 — Supplementary information 3 [file 41598_2020_70730_MOESM3_ESM.docx]

| **Gene** | **GenBank accession no.** | **Primer sequence** |
| --- | --- | --- |
| *S29* | NM_009093 | F GGAGTCACCCACGGAGTTCG |
|  |  | R: GGAAGCAGCTGGCGGCACATG |
| *Col6a1* | NM_009933 | F: GACAGTCTCCAGGAAGGT |
|  |  | R: GACAGTTGAGTTGACCAGTT |
| *Opn* | NM_009263 | F: CGATGATGATGACGATGGAG |
|  |  | R: GAGGTCCTCATCTGTGGCAT |
| *Ocn* | NM_031368.5 | F: GTCACTCTGTCCTCTTGGTA |
|  |  | R: GCTGCTGTGACATCCATACTTGC |
| *Oscar* | NM_175632 | F: ACTCCACCAGATACTCATTCT |
|  |  | R: AGTCACTAACATTAGCTGAACAT |
| *Cstk* | NM_007802 | F: TGGCTCGGAATAAGAACAAC |
|  |  | R: AAGGAAGGAATCTGAGAAGAGA |
| *Tnfα* | NM_013693 | F: AGGACTCAAATGGGCTTTC |
|  |  | R: AGGTCTGAAGGTAGGAAGG |

**Supplemental Table 1**. List of primers used for Real-Time RT-PCR. F: forward/sense; R: reverse/antisense
