# Supplementary material for: Collagen VIα2 chain deficiency causes trabecular bone loss by potentially promoting osteoclast differentiation through enhanced TNFα signaling
Source: Sci Rep. 2020 Aug 13;10:13749. doi: 10.1038/s41598-020-70730-7 (PMC7426410; doi:10.1038/s41598-020-70730-7)
Supplement: Supplementary file 4 — Supplementary information 4 [file 41598_2020_70730_MOESM4_ESM.docx]

**Supplemental Table 2.** Top 5 upstream regulators. Differentially expressed genes (total 1107, Down: 466, Up: 641) from DESeq2 were applied to Ingenuity pathway analysis (IPA) (Qiagen). IPA identified the top 5 upstream regulators including TNFa.
